# Newly Designed and Experimental Test of the Sediment Trap for Horizontal Transport Flux

**DOI:** 10.3390/s22114137

**Published:** 2022-05-30

**Authors:** Tao Liu, Zihang Fei, Lei Guo, Jiarui Zhang, Shaotong Zhang, Yan Zhang

**Affiliations:** 1 Shandong Provincial Key Laboratory of Marine Environment and Geological Engineering, Ocean University of China, Qingdao 266100, China; ltmilan@ouc.edu.cn; 2 Laboratory for Marine Geology, Pilot National Laboratory for Marine Science and Technology, Qingdao 266061, China; 3 College of Environmental Science and Engineering, Ocean University of China, Qingdao 266100, China; fzh2988@stu.ouc.edu.cn (Z.F.); zhangjr@stu.ouc.edu.cn (J.Z.); 4 Institute of Marine Science and Technology, Shandong University, Qingdao 266237, China; 201894900036@sdu.edu.cn; 5 College of Marine Geosciences, Ocean University of China, Qingdao 266061, China; shaotong.zhang@ouc.edu.cn

**Keywords:** marine suspended sediments, transport processes explanation, accompanying transport fluxes analytic formula, time-series vector

## Abstract

The transport processes of marine suspended sediments are important to the material cycle and the shaping of seafloor topography. Existing sediment monitoring methods are limited in their use under high concentration conditions, and are not effective in monitoring and capturing sediment in 3D directions, and there is an inability to accurately explain sediment transport processes. To infer the transport process of suspended sediments, this study proposed a time-series vector in situ observation device. An accompanying time-series analytic method was developed for sediment transport fluxes. The correlation between the internal and external flow velocities of the capture tube was established through indoor tests, and then the applicability of the device was verified by the correlation between the theoretical capture quality and the actual capture quality, and the analytic formula of the flux was refined. The proposed observation technique can be used for in situ long-term observation and sampling of marine suspended sediments under conventional and even extreme sea conditions, achieving accurate time-series suspended sediment capture and high-resolution transport flux analysis. The technique thus provides a more effective means for scientific research into the dynamics of seafloor sedimentation, the mechanisms of ocean carbon sinks, and the processes of the carbon cycle.

## 1. Introduction

Large river estuaries transport large amounts of sediment to the ocean each year [1,2]. These marine sediments are deposited far from the estuary and distributed deep into the oceans. Their transport processes and pathways have important effects on the environment and evolution of estuarine and marine landscapes [3,4].

Sediment transport mechanisms and flux changes are an important part of the current international sediment source-to-sink research program, where riverine sediments undergo complex dynamics in the seafloor boundary layer and generate multiple types of seafloor, sediment flow, and dispersion forms [5,6,7,8,9,10]. Research into the resuspension and transport of sediments due to ocean dynamics usually relies on in situ observations synchronized at multiple stations, which are costly and unable to describe the transport process. The mechanisms of ocean carbon sinks and the carbon cycle have received international attention with the proposal of the concept of “carbon peaking and carbon neutrality.” Coastal areas bury 80% of continental organic carbon and account for one quarter of global ocean primary production. When ocean sediments are re-suspended, the buried carbon particles are re-released into seawater. At a local or regional scale, the geomorphological dynamics of river basins and coastal zones resulting from erosion, transport and deposition of particles, and the associated transport of nutrients and contaminants, require investigating sediment transport. The lack of effective means and methods for observing carbon particle resuspension has made it difficult to calculate the carbon fluxes released from the seafloor to seawater, which has hindered the development of research on global ocean carbon sink mechanisms [11,12,13,14].

Methods commonly used in marine sediment observation include in situ sampling, satellite remote sensing, moored sediment traps, and in situ observation from seated platforms [15,16,17].

In situ sampling can directly obtain field samples and accumulation status, which can provide an overall understanding of the concentration of suspended sand in a certain area and facilitate direct analysis of the spatial distribution of sediments with real reliability. However, the method is cumbersome and inefficient and cannot be monitored continuously in real time; only scattered turbidity data can be obtained, and peak data may be lost in the observation, which does not allow for detailed study of the dynamic processes of suspended sediment [18].

Remote sensing technologies can provide global coverage of surface land and water regions over the whole Earth’s surface; hyperspectral remote sensing as a proven technology can be used in identifying and mapping spectrally active materials in applications. Numerical models gained an acceptable level of representativeness to be new a model involved in cohesive sediment transport process studies and hydro- and geo-sciences phenomena [19,20,21,22,23]. The sediment transport model can be calibrated and validated by observed hydrological data and remote sensing results, to investigate the impacts of the sediment, including sediment movement, sediment source, dispersion range, transport direction, and sand content interpretation of water bodies [24,25,26]. However, the numerical modeling of sediment transport is prevented from achieving a high level of predictability by the difficulties in estimating poorly known model parameters. Furthermore, the satellite remote sensing is only suitable for macroscopic monitoring of large areas; it has limited ability to estimate the suspended sediment concentration at certain water depths, and it cannot accurately monitor the sediment transport process in the benthic boundary layer, making it difficult to further investigate the causal factors and mechanisms of sediment transport [27].

Anchor sediment traps are a commonly used direct measurement tool, and they were first used in studies of seafloor organic matter transport and ocean carbon sinks [28]. Sediment traps are divided into vertical sediment traps and horizontal sediment traps. The vertical sediment trap is capable of in situ collection of naturally deposited particulate matter within a certain area of the water. In the field of marine geology, sediment traps are commonly used to carry out research on sediment dynamics and erosion and siltation processes. By collecting sediments that settle from top to bottom, a sediment deposition sequence is formed, and subsequently physicochemical indices, such as particle size composition, mineral composition, and chemical composition, are obtained in indoor experiments. These conventional traps with upward openings can be used to study the nature of settling sediment debris with multi-layered ocean fluxes, but they cannot be used to examine the impact from horizontal sediment transport [29,30]. In terms of horizontal traps, such as a pressure-difference bedload sampler, portable traps, streamer sediment trap, and cage-shaped sediment trap can capture sediments that are transported horizontally. However, conventional traps that can perform horizontal sediment measurements fail to resolve the temporal and spatial variability of sediment transport fluxes and cannot effectively analyze the transport mechanisms of marine suspended sediments [31,32,33,34]. 

As the most important indirect estimation method of the sediment transport process, the sit-down observation platform allows the researcher to choose the type of instrument on board, such as an optical back scattering sensor (OBS), acoustic back scattering sensor (ABS), laser particle analyzer (LPA), or acoustic doppler current profiler (ADCP). The researcher can indirectly estimate the suspended sand concentration through the acoustic and optical equipment on board, but this method cannot be used to directly capture and analyze sediment samples [35,36,37,38].

The following Table 1 is a comparison of the mainstream monitoring techniques for sediment transport processes.

From field investigations, it has been found that some extreme sea conditions such as storm surges, which carry large amounts of sediment deposits and produce high concentrations of mudflows on the seafloor, cause significant erosion of the shoreline and play a major role in controlling the transport of seafloor sediments [39,40,41,42]. These extremes have greatly increased the difficulty of accurately observing sediment transport and affected the analysis of sediment transport mechanisms and stratigraphic records [43,44,45]. New horizontal traps are limited to shallow areas or areas with low flow velocities, and so they cannot be used for observation in extreme environments. The seated-bottom observation platform is ineffective in extreme environments with extremely high turbidity, complex species, and rapid changes, such as storm surges, due to the restrictive range and operating principle of various observation instruments.

No technical means exist to effectively combine the advantages of a seated-bottom observation platform and a sediment trap, which would allow for time-series vector observation of sediment transport at high suspended sand fluxes and for the capture of sediment samples for indoor analysis. This paper proposes a time-series 3D sediment trap for monitoring transport fluxes (3D trap). It is an in situ sediment trap for marine suspended sediments. The study also establishes an analytic formula for a multi-level sediment transport flux time series based on flow velocity and suspended sand concentration.

The 3D trap facilitates long-term in situ monitoring of marine suspended sediment transport fluxes under extreme sea conditions. It is therefore complementary to the current mainstream indirect and low-concentration observation methods, such as acoustic and optical methods. Complemented by indoor experimental analysis of sediment samples obtained at each moment, the 3D trap enables the physical and chemical properties of sediments to be obtained to complete in situ, long-term, 3D, and dynamic observation of marine suspended sediment transport mechanisms. It can also reveal the spatial and temporal distribution characteristics of marine sediment transport processes and their controlling factors. Moreover, it can be used to explain the dynamic evolution mechanism and characteristics of transported sediments in a quantitative manner. Furthermore, it gives the key dynamics of scientific research processes such as erosion and siltation, material transport, and elemental cycling, and so on. It also provides a new perspective and technical means for the study of sediment from source to sink and the reduction of the marine carbon sink mechanism.

## 2. Design of the 3D Trap

### 2.1. Structural Design

**Figure 1:**
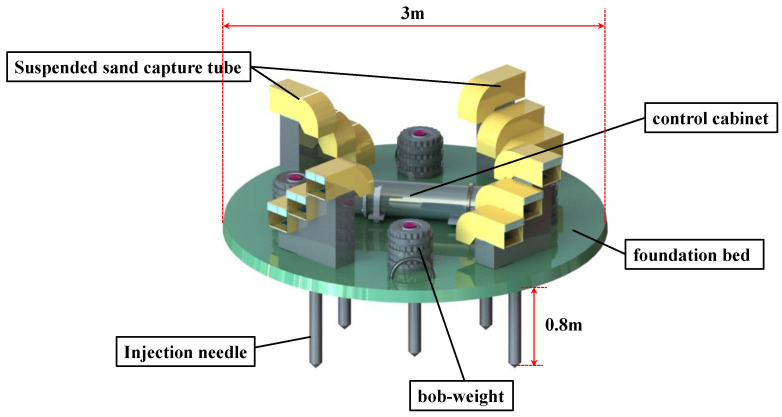
Schematic of the 3D trap.

The 3D trap is different from vertical sediment traps. Its main structure consists of a sediment-trapping system, observation system, control cabin, platform frame, and subsidence-compensation system. The bottom of the 3D trap is a circular sit-down observation platform with a penetrating pin that can be inserted into the seabed after being placed on the seafloor to ensure the stability of the whole system and facilitate sediment capture from multi-directional transport sources on the seafloor. The Schematic of 3D trap is shown in the Figure 1.

As shown in Figure 1, the 3D trap includes three suspended sand traps with equal depth gradient distribution in all four directions of the observation base. In the center of the base is a control module, which includes an acquisition controller, nine-axis sensing components (triaxial accelerometer, triaxial gyroscope, triaxial magnetometer), a wave and tide meter, and a battery pack for storing and reading monitoring data or sensing the attitude and orientation of the base. The weight of the connection between the base and the suspended sand trap is adjustable. The trap is equipped with an adjustable weight and center of gravity. It relies on the operating vessel for deployment. 

**Figure 2:**
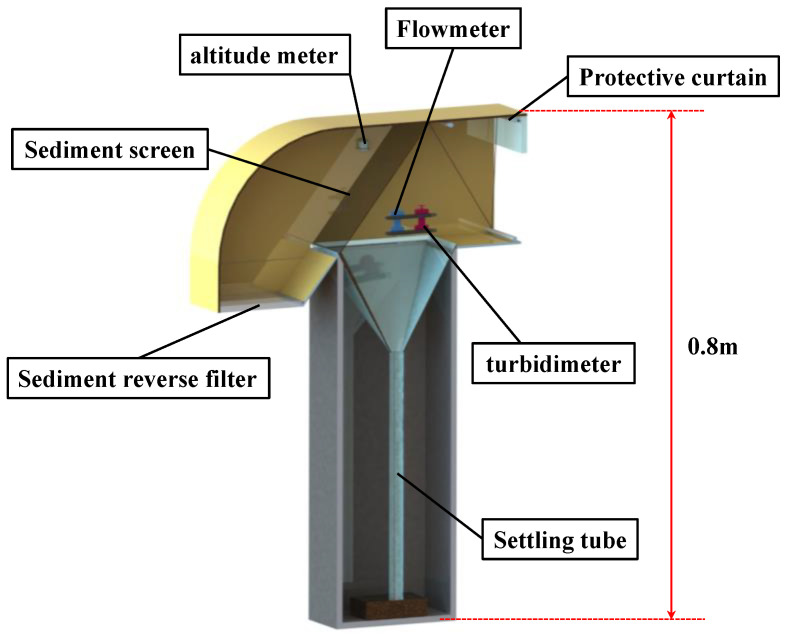
Structure of the capture tube.

Figure 2 shows the suspended sand-capture pipe, including a water flow pipe and a sinker pipe. The front end of the water flow pipe has a horizontal water inlet; the rear end has a vertical downward outlet. The vertical outlet was set downward to ensure that the turbidity flow does not return by gravity after flowing through the capture tube, and the vertical capture tube wall blocks the reverse turbidity flow so that it does not back up the capture tube. The middle of the water flow pipe is fitted with a sediment-filter screen tilted in the direction of the inlet. The sinker is fixed vertically below the screen, sealed at the bottom and open at the top, and connected to the water flow pipe.

### 2.2. Sensors

The sensors in the 3D trap include a current meter and a turbidimeter. The current meter is used to monitor the average velocity vector values of the flow field inside the device. Other functions have not yet been considered. There are various requirements for the current meter: it needs to be relatively small and portable so that it can be installed inside the device; it needs to have a self-contained monitoring system to store long-term in-situ monitoring data; it needs to be high-pressure resistant so that it can be placed in the subsea boundary layer; and it must be able to provide continuous monitoring. It is proposed that the device use the JFE brand INFINITY-EM small self-capacitating electromagnetic current meter. Its indicators meet the monitoring requirements and have high suitability for the whole device.

The turbidimeter is used to monitor the turbidity of sediment carried by the internal flow field of the device. Other functions have not yet been considered. The turbidimeter needs to be small and portable; it must include a self-capacitating monitoring system; it should be resistant to high pressure; and it should provide monitoring continuity. It is proposed that the device use the RBR brand RBRsolo³ Tu miniature turbidimeter. Its indicators meet the monitoring requirements and have a high fit for the whole device.

These sensors will continue to be updated, and the sensors will be continually selected for optimal solutions to ensure that the requirements of the in-situ long-term monitoring device are met. 

### 2.3. Analytic Formula for Sediment Transport Fluxes

**Figure 3:**
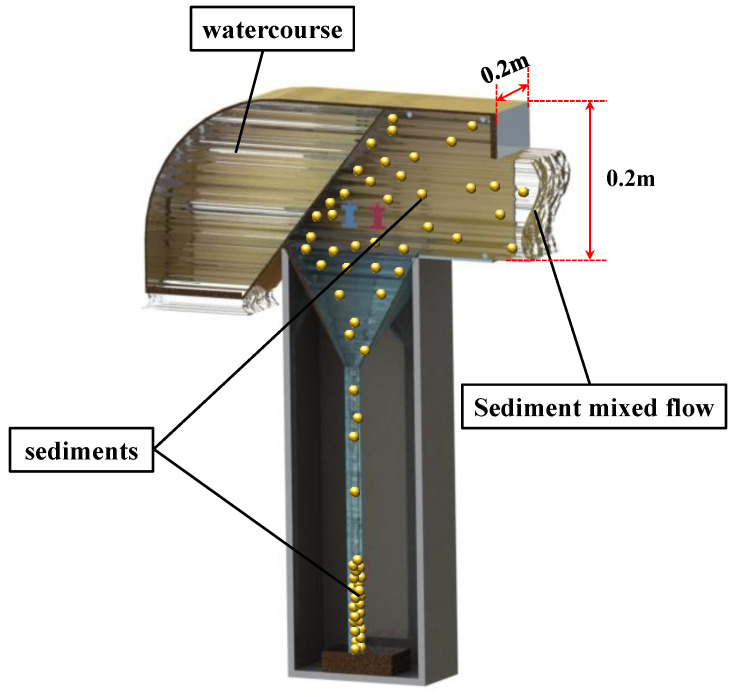
Working principle of the capture tube.

Based on the catcher design, the sediment screen can intercept the sediment that is flowing through the flow pipe and toward the settling pipe for deposition by the force of gravity. The principle of action is shown in Figure 3.

Each direction of the trapping system includes three trap ports at different elevations. These are used to collect suspended sand from different layers in the respective direction. The suspended sediment transported by the water through the observation point in different directions at different levels is then captured in a 3D manner.

With a current meter and a turbidimeter inside the current tube, the suspended sediment concentration $SSC_{\left(d,h,i\right)}$ and velocity $V_{c\left(d,h,i\right)}$ of the water flowing through the current tube can be continuously collected and recorded during the observation period. 

An analytic formula can be established for the sediment transport flux based on flow velocity, the suspended sand concentration at the trap mouth, and the total amount of sediment captured by the trap:

(1) $$Q_{\left(d,h,t\right)}^{}=\frac{1}{S\times k}\sum_{i=t-k}^{t}V_{c\left(d,h,i\right)}\times SSC_{\left(d,h,i\right)}$$

where *d* is the sediment transport direction, *h* is the depth from the bottom, *t* is the corresponding moment, *k* is the time interval, *S* is the opening area of the suspended sand-capture tube, *V_c_* is the flow rate in the tube, and *Q* is the flux of sediments at different *d*, *h*, *t*.

Because this is a new method proposed by the author, the method can be considered applicable from low-flow, low turbidity to high-flow, and high turbidity waters where laterally transported sediments can be captured by the device. In addition, the use of the analytical method is based on certain assumptions, as shown in the assumptions in Table 2.

### 2.4. Research Methodology

**Figure 4:**
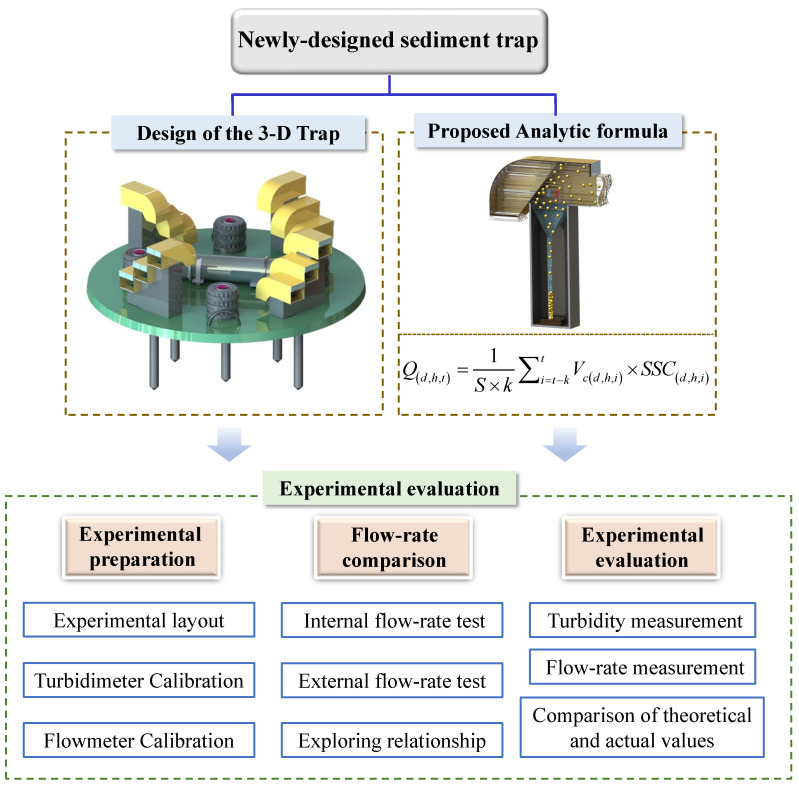
Flowchart of the methodology.

A flowchart of the methodology is given here to see the overall research idea more clearly. As shown in the Figure 4.

## 3. Experimental Evaluation

To test the feasibility of the device’s design and analytic formula, laboratory flume simulation experiments were conducted. The aim of these experiments was to establish and refine the quantitative relationship between sediment flux analysis (based on flow rate and suspended sand concentration) and captured sediment samples to verify the device. The tests mainly verified the rationality of the structure of Figure 3, and when considering the feasibility of the complete unit, only additional verification of the effect of the overall unit on the flow field is needed.

After the experimental scenario was set up with the relevant equipment, the calibration of the relationship between turbidity and suspended sand concentration was performed to determine the conversion between the turbidimeter parameters and the experimental parameters. Since the internal flow velocity of the equipment could not be measured directly in the experimental test phase, the external iso-gradient flow velocity was measured instead. Given the influence of the equipment structure on the difference between the internal and external flow fields, the quantitative relationship between the two was verified by a water tank test. After determining this relationship, the test was continued by adjusting the flow rate and capturing several sets of samples. The capture mechanism of the water tank device was verified by checking the sample volume, and the flux resolution equations were refined by comparing the captured sample weights with the theoretical calculated values. During this phase, indoor model tests were performed to ensure that parameters such as the capture tube’s height-to-diameter ratio, roll-up area, mesh tilt, mesh aperture, and head-on angle were consistent. 

### 3.1. Experimental Layout

**Figure 5:**
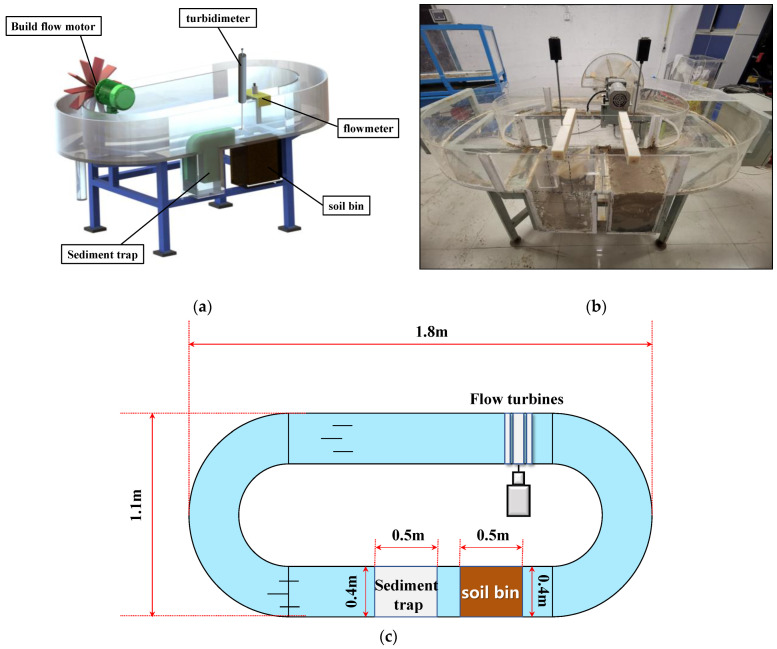
(**a**) Main structural design of the water flume; (**b**) Description of what is contained in the second panel; (**c**) Flume diagram.

An indoor multi-stage recirculating flow-generating tank was used (Figure 5) [46]. It was made of acrylic material with a thickness of 1 cm, and the joints were reinforced with steel plates and coated with epoxy resin. The tank had dimensions of 180 cm (L) × 110 cm (W) × 50 cm (H). Inside the tank were a flow-generation system and a scouring system. The flow-generation system consisted of a rotating motor and its flow-generation paddle, which can control the simulated scouring flow rate (0–50 cm/s). The scouring system consisted of a flume channel, a capture-tube placement tank, and an earth tank. Both tanks were 50 cm (L) × 40 cm (W) × 50 cm (H), and the opening area of the capture tube was 8 cm × 8 cm.

The measurement system consisted of a portable flowmeter and a turbidimeter (RBR Canada/XR-620CTDTu). The measurement sensor of the flow meter and the OBS probe of the turbidity meter were fixed directly above the bed of the sink in front of the opening of the capture tube. They were used to measure the real-time changes of flow rate and turbidity outside the capture tube. 

The equation proposed by Qin [47] for calculating the starting flow rate for each particle size of non-uniform sand is as follows:

(2) $$\frac{U_{c}}{\sqrt{\frac{\gamma_{s}-\gamma }{\gamma }gD}}=0.963\sqrt{0.67+\frac{D_{m}}{D}}{\left(\frac{h}{D_{90}}\right)}^{1/6}$$

where $U_{c}$ is the starting speed of sediment particles, $\gamma_{s}$ is the unit weight of sediment particles, γ is the unit weight of water, D is the sediment particle size, $D_{m}$ is the average particle size, $D_{90}$ is the 90% of the particle size distribution number, and h is the water depth.

After a series of processes such as drying in a dryer, sieving soil in a vibrating sifter, weighing on an electronic scale, and analysis by a particle size analyzer, we finally obtain standard soil particles with an average particle size of 0.21 mm in the range of 0.15 to 0.25 mm. Since the particle size floating range was small at this time, the difference relationship between its concentration inside and outside the capture tube can be ignored for the time being.

The water depth in the water tank was set at 13 cm. The speed of the flow in the water tank was controlled between 10 cm/s and 40cm/s. The filter mesh aperture was set at 100 mesh (0.15 mm), and so the particle size was between 0.15 mm and 0.25 mm. The test was carried out with five groups of flow rate of 15 cm/s, 20 cm/s, 25 cm/s, 30 cm/s, and 35 cm/s, and the parameters are set as shown in Table 3.

### 3.2. Calibration of Turbidity in Relation to Suspended Sand Concentration

**Figure 6:**
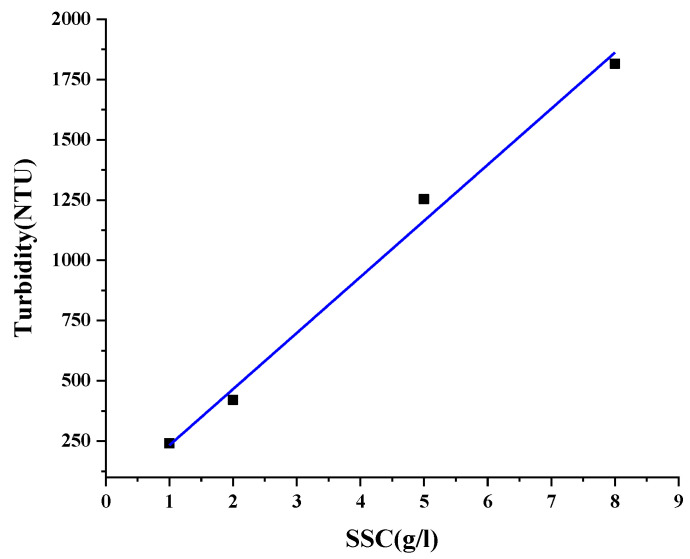
Conversion curve of turbidity and suspended sand concentration.

The equation to convert turbidity to suspended sand concentration was obtained by an indoor calibration experiment. First, five groups of different masses of particulate matter were selected and added to a fixed volume of water with sufficient stirring to prepare a sequence of suspensions with increasing concentrations. The sensor was connected to the matching Ruskin software via a USB cable, and the acquisition time and frequency of the sensor were set up. The software was set to record the turbidity change of the water body with a sampling frequency of 3 s each time. The turbidity of the suspension was measured by using a turbidimeter to obtain four sets of corresponding data, and the data obtained were compared with the known concentration of suspended sand, the results are shown in Figure 6. A linear relationship between SSC and NTU was currently know [46], and the relationship between the two is as follow:

(3) NTU=(232.8±6.6)SSC

### 3.3. Internal and External Flow-Rate Comparison Test

Given the small diameter of the capture tube, the internal capture screen, and the bend structure at the end, the external flow field changes negligibly when flowing through the capture tube. A screen of suitable pore size was installed at an angle inside the capture tube to ensure that all sediments were trapped. There was a large difference between the internal and external flow rates, which causes a serious deviation from the actual catch in the transport flux analysis by substituting external flow rates. A set of experiments was therefore conducted to compare the internal flow velocity of the device with the external flow velocity.

A single-point mechanical LB50-1C rotating cup flow meter was used to design and process an opening at the top of the capture tube model, and the flowmeter measuring probe was placed directly above the sink tube and sealed with an acrylic plate clamp and waterproof tape to measure the flow velocity of the water body above the sink tube. 

**Figure 7:**
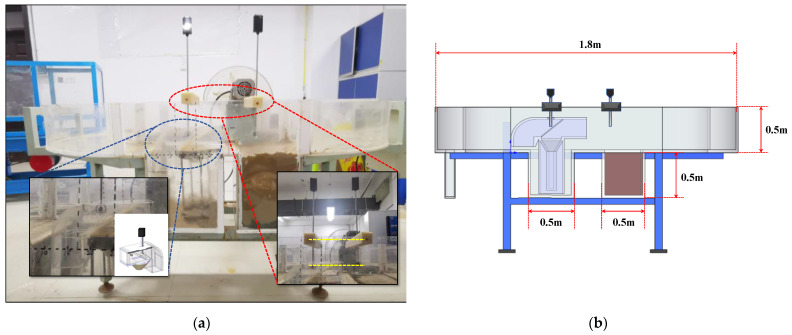
(**a**) Field test chart; (**b**) Test schematic.

The experiments involved the following steps: Start the annular water flume, adjust the flow rate in the flume by adjusting the speed of the flow-making paddle, and record multiple sets of internal and external readings of two flowmeters at the same time to obtain the relationship between the two, as shown in Figure 7. The relationship after fitting is as follow, R^2^ = 0.994:

(4) $$V_{\mathrm{out}}=\left(0.0643\pm 0.0034\right)V_{\mathrm{in}}+\left(-6.0377e^{-4}\pm 1.0089e^{-4}\right)V_{\mathrm{in}}{}^{2}$$

**Figure 8:**
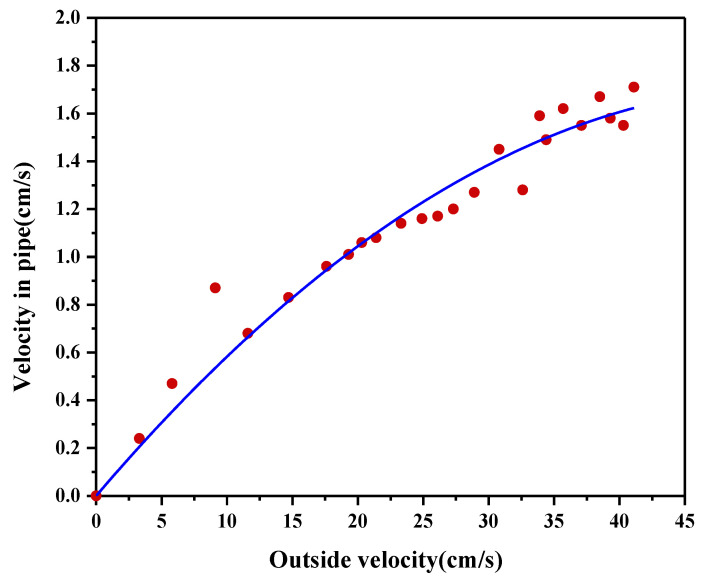
Correlation between internal and external flow rates.

Figure 8 shows that the internal and external flow velocities are positively correlated in general, and the difference between them gradually increases as the flow velocity increases. The modified indoor test confirms that the flux equation is as follows:

(5) $$Q_{\left(d,h,t\right)}^{}=\frac{1}{S\times k}\sum_{i=t-k}^{t}[\left(0.0643\pm 0.0034\right)V_{{}_{c\left(d,h,i\right)}}^{\prime }+\left(-6.0377e^{-4}\pm 1.0089e^{-4}\right)V_{{}_{c\left(d,h,i\right)}}^{\prime }{}^{2}]\times SSC_{\left(d,h,i\right)}$$

$V_{{}_{c\left(d,h,i\right)}}^{\prime }$ is the external flow rate of the device; that is, the actual measured flow rate of the test.

### 3.4. Experimental Test of the Calibrated Sediment Transport Flux Formula

After all the preparatory tests were completed and the expected results were obtained, the water tank test was conducted to verify and refine the analytic formula:

(6) $$Q_{\left(d,h,t\right)}^{}=\frac{1}{S\times k}\sum_{i=t-k}^{t}V_{c\left(d,h,i\right)}\times SSC_{\left(d,h,i\right)}$$

**Figure 9:**
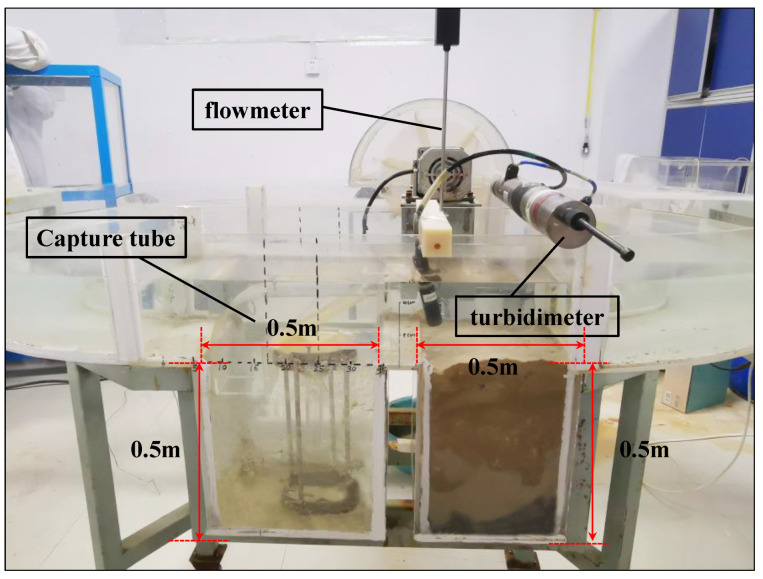
Flux analysis test.

The flume was laid out and the position of the trap was adjusted so that the head-on flow of the trap tube faced the flow field. The sink tube was placed inside the flume at the rear of the soil flume. The flow meter was fixed in front of the mouth of the trap tube by a fixed device so that the rotor was located at the center of the trap tube. The turbidimeter was set in front of the flow meter, and also positioned at the center of the trap tube. The experimental set up is shown in Figure 9. 

After the tank was filled with water, the flow-making paddle was started. The flow rate at the location of the capture tube was controlled at 15 cm/s, 20 cm/s, 25 cm/s, 30 cm/s, and 35 cm/s. A relatively constant state of turbidity was maintained to keep the flow rate constant for 10 min. The moment when the specified flow rate started and ended was recorded. The data from the turbidimeter is exported, the turbidimeter display for the corresponding time period is found by the previously recorded start moment of the flow meter, and the integration is performed for that time period. At the end of each collection, the capture tube was taken out and left to stand for 3 min, and the tube was then removed to collect the deposited particles. Three sets of parallel control tests were set up at each flow rate to obtain 15 sets of samples. All samples were filtered, dried, and weighed on an electronic tray to obtain the weight of fifteen sets of samples at five flow rates.

**Figure 10:**
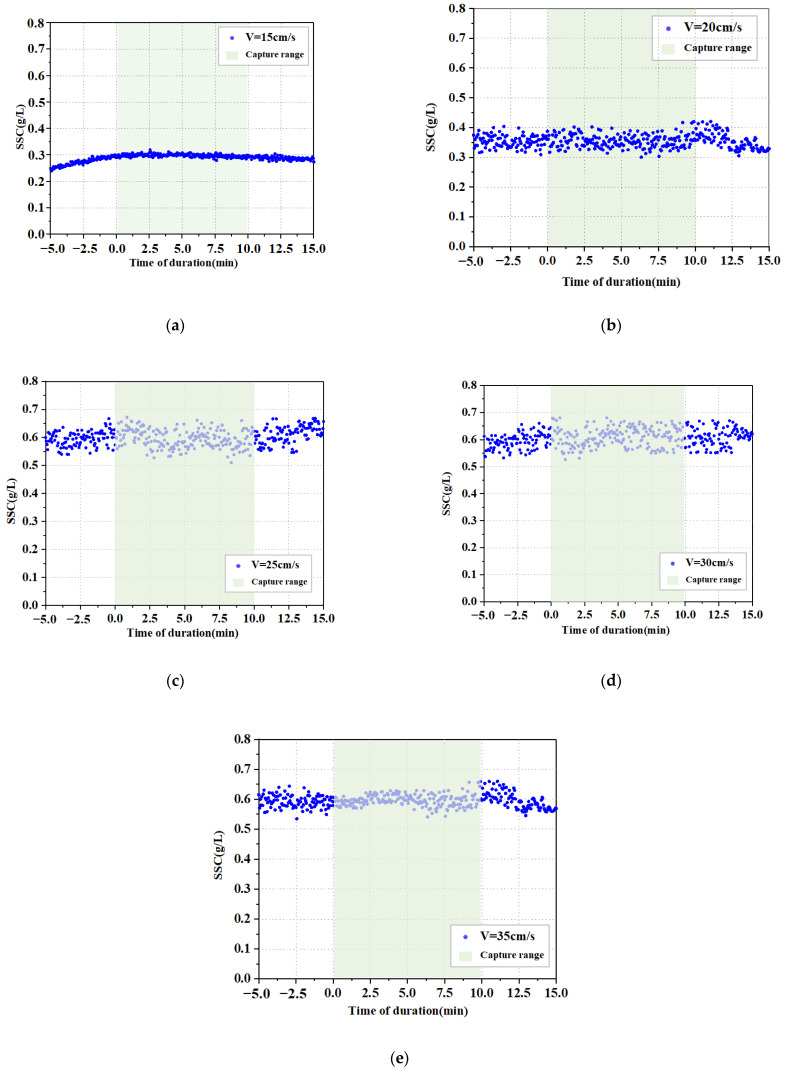
Variation of suspended sand concentration at each flow rate: (**a**) 15 cm/s; (**b**) 20 cm/s; (**c**) 25 cm/s; (**d**) 30 cm/s; (**e**) 35 cm/s.

The turbidimeter was connected to the computer to upload the data for analysis. One set of results is shown in Figure 10, where each group of data is collected from 0 to the end of 10 min time. We calculated the turbidity for the corresponding time period and obtained the total turbidity value for that time period.

**Figure 11:**
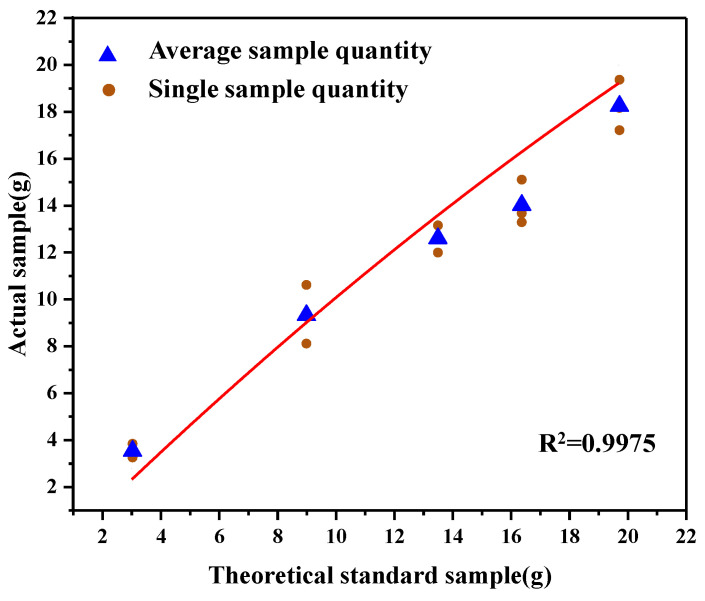
Correlation between theoretical capture and actual capture.

After measurement, the theoretical value was compared with the actual catch value, and the result is shown in Figure 11. The correspondence is as follow, R^2^ = 0.9975:

(7) $$y=(1.059\pm 0.124)x-(0.008\pm 0.007)x^{2}$$

The theoretical capture quality corresponds well with the actual catch value, thus confirming the validity of the analytic formula for sediment transport fluxes. 

### 3.5. Discussion

The article first proposes a device, and then gives the structure diagram and design idea of the device, as well as the supporting analytic: Formula (1) for sediment transport fluxes. The conversion relationship between turbidity and suspended sand concentration of the turbidimeter used was carried out by indoor tests, and the relationship is seen in Formula (4). Finally, the validation test of the analytic formula was carried out, and the analytic Formula (7) was verified and refined by comparing the theoretical capture quality calculated by the analytical flux formula with the actual capture quality.

In the process of field application, the device needs to be used in extensive waters because of its large size, which will have a serious impact on small space waters; since the device can only capture sediment in a horizontal direction and in a fixed direction, it is necessary to first detect the capture area and then deploy the device according to the needs of engineering/research. Since the capture of the device relies mainly on the interception of the screen, it is impossible to capture all the sediments, so the selection of the screen needs to be based on the detection results and engineering/research needs; since the capture capacity of the device is limited, the protective curtain needs to be adjusted continuously during the capture process to control the cross-sectional area of the trap opening, and the transport flux is adjusted proportionally.

The 3D trap is usually placed in two locations; one can be placed in the estuary, straits and other high velocity/turbidity areas, in order to achieve the effective use of the device; and one at an interval of more than 4000 m in the open water for deployment [48]. This interval reference triangular mesh grid division with local mesh refinement can effectively carry out the monitoring and feedback of regional sediment transport while ensuring the feasibility of deployment and recovery.

Sediment is an important component of erosion and siltation, and a 3D trap can be used to monitor the erosion and siltation in a marine environment and a lake and marsh area, so as to prevent and control engineering disasters in advance and reduce engineering economic losses caused by erosion. Many sediments belong to land-based debris and marine pollutants, which have serious impact on marine ecological environment. A 3D trap can be used in the field of environmental protection to monitor sediments and in situ invert their transport pathways and transport mechanisms, so as to control the sediment pollution and reduce the loss of environmental benefits caused by sediment transport. A 3D trap can also be used in the field of carbon sink to analyze the process of sediment resuspension and promote the research of carbon sink mechanisms.

In future applications, the steering module and the corresponding algorithm will be added to the device to achieve the function of sediment capture by steering with the current. The device will add a sedimentation compensation module to ensure the relative height of the sedimentation tube is constant through the adaptive adjustment of the height of the device, which could increase the accuracy of monitoring, and the amount of sediment captured can be more accurately determined by adding turbidity meters both before and after the capture tube. 

## 4. Conclusions

This paper proposes a device called the 3D trap based on marine suspended sediment. The device is equipped with an internal flow meter and turbidimeter and a settling tube for processing captured sediment. When the particles enter the trap tube, they are settled in the trap tube due to the action of the filter, and the sample taken is the actual catch. In addition, a multi-level sediment transport flux time-series analytic formula was developed based on flow rate and suspended sand concentration. The analytic formula uses real-time counting by the turbidimeter and a flow rate meter to derive the theoretical sediment capture quality. In addition, indoor tests were conducted to investigate the relationship between the external flow velocity and the internal flow velocity of the structure, and the flux analysis formula was refined based on the test results. Finally, the correlation between the actual catch and the theoretical catch calculated by the refined analytic formula was determined experimentally. The refined analytic formula for in situ sediment transport fluxes based on in situ capture of suspended marine sediments was shown to be compatible with the device.

(1) This study proposes a sediment capture and transport process time-series vector observation device that can be used to conduct in situ long-term monitoring and process reduction of lateral sediment transport flux under normal and even extreme sea conditions. It can also capture sediment samples, providing a more effective technical means for frontier scientific research into seafloor sediment dynamics and material cycling processes.
(2) A set of analytic formulas for sediment transport fluxes based on flow velocity and suspended sand concentration is established for the time-series vector observation device. The non-negligible difference between external flow velocity and internal flow velocity caused by the structure of the device is determined and the conversion equation is proposed.
(3) The reliability of the analytical method for transport fluxes is verified, and the analytic formula is refined through indoor experiments. The results verify the effectiveness of the capture mechanism, which lays the foundation for the application of the time-series vector observation device for sediment capture and transport processes.

The device can be used to monitor and capture sediment transport fluxes under conventional and even extreme sea conditions. It can also be used to analyze and explain sediment transport processes with high accuracy, providing a novel way to study sediment resuspension caused by oceanic processes, in addition to being a more effective technical means for scientific research into the dynamics of seafloor sedimentation, ocean carbon sink mechanisms, and carbon cycle processes.

## Figures and Tables

**Table 1:** Comparison of mainstream monitoring technologies for sediment transport processes.

Monitoring Technology Category	Range	Precision	Timeliness	Samples	Platform
sample measurement	No restrictions	low	low	Single sample	yes
OBS	low	high	high	no	yes
ABS	low	high	high	no	yes
ADCP	low	high	high	no	yes
satellite remote sensing	No restrictions	low	high	no	yes
sediment traps	No restrictions	high	high	Complex samples	no

**Table 2:** Assumptions table.

Factors	Assumptions
Flow Rate	Controlled by protective curtain within measurable interval
Turbidity	Continuous inside and outside the capture tube
Space	Plenty enough to ensure that the device does not seriously affect the flow field
Sediment particle size	Most particle sizes larger than the selected screen aperture
Capacity	Sufficient for long-term in situ monitoring

**Table 3:** Control of experimental conditions.

Experimental Factors	Experimental Setup				
Speed (cm/s)	15	20	25	30	35
Particle size (mm)	0.15~0.25	0.15~0.25	0.15~0.25	0.15~0.25	0.15~0.25
Screen pore size (mm)	0.15	0.15	0.15	0.15	0.15
Depth (cm)	13	13	13	13	13

## Data Availability

Not applicable.
